# Postoperative resveratrol administration improves prognosis of rat orthotopic glioblastomas

**DOI:** 10.1186/s12885-018-4771-1

**Published:** 2018-09-03

**Authors:** Xue Song, Xiao-Hong Shu, Mo-Li Wu, Xu Zheng, Bin Jia, Qing-You Kong, Jia Liu, Hong Li

**Affiliations:** 1 Liaoning Laboratory of Cancer Genetics and Epigenetics and Department of Cell Biology, College of Basic Medical Sciences, Dalian Medical University, Dalian, 116044 China; 2 South China University of Technology School of Medicine, Guangzhou, 520006 China

**Keywords:** Resveratrol, Lumbar puncture, Postoperative chemotherapy, Rat orthotopic glioblastoma, STAT3 signaling

## Abstract

**Background:**

Although our previous study revealed lumbar punctured resveratrol could remarkably prolong the survival of rats bearing orthotopic glioblastomas, it also suggested the administration did not completely suppress rapid tumour growth. These evidences led us to consider that the prognosis of tumour-bearing rats may be further improved if this treatment is used in combination with neurosurgery. Therefore, we investigated the effectiveness of the combined treatment on rat orthotopic glioblastomas.

**Methods:**

Rat RG2 glioblastoma cells were inoculated into the brains of 36 rats. The rats were subjected to partial tumour removal after they showed symptoms of intracranial hypertension. There were 28 rats that survived the surgery, and these animals were randomly and equally divided into the control group without postoperative treatment and the LP group treated with 100 μl of 300 μM resveratrol via the LP route. Resveratrol was administered 24 h after tumour resection in 3-day intervals, and the animals received 7 treatments. The intracranial tumour sizes, average life span, cell apoptosis and STAT3 signalling were evaluated by multiple experimental approaches in the tumour tissues harvested from both groups.

**Results:**

The results showed that 5 of the 14 (35.7%) rats in the LP group remained alive over 60 days without any sign of recurrence. The remaining nine animals had a longer mean postoperative survival time (11.0 ± 2.9 days) than that of the (7.3 + 1.3 days; *p* < 0.05) control group. The resveratrol-treated tumour tissues showed less Ki67 labelling, widely distributed apoptotic regions, upregulated PIAS3 expression and reduced p-STAT3 nuclear translocation.

**Conclusions:**

This study demonstrates that postoperative resveratrol administration efficiently improves the prognosis of rat advanced orthotopic glioblastoma via inhibition of growth, induction of apoptosis and inactivation of STAT3 signalling. Therefore, this therapeutic approach could be of potential practical value in the management of glioblastomas.

## Background

Glioblastoma multiforme (GBM) is the most common primary brain malignancy and is associated with an extremely poor prognosis because of its highly aggressive growth and the difficulty of radical resection [1, 2]. Consequently, the majority of GBM patients receiving standard-of-care postoperative radiation and chemotherapy die within 12 months [3]. Surgery is the first choice to treat GBMs, and resection can remove up to 78% of the tumour mass. However, the residual cancer cells can infiltrate into brain tissue and must be treated with adjuvant chemo- and/or radio-therapy [4, 5]. Normal brain tissue is sensitive to radiotherapy and the frequent resistance of glioblastoma cells to conventional anticancer drugs such as temozolomide (TMZ) are major challenges in GBM-oriented adjuvant therapies [6]. Thus, identifying more effective and less toxic anticancer agents that are able to penetrate the blood–brain barrier are critically needed for the management of glioblastomas.

Accumulating in vitro data reveal that resveratrol, a natural polyphenoal compound, exerts inhibitory effects on human brain malignancies including medulloblastoma and glioblastoma cells [7–9]. More importantly, resveratrol is able to cross the blood–brain barrier and has limited toxic effects on normal brain cells [10]. However, resveratrol is quickly metabolized in vivo upon absorption and this leads to very low bioavailability [11]. We have administered resveratrol via lumbar puncture (LP) to overcome the bioavailability limitation. Our results revealed a remarkable increase of resveratrol in rat brains and prolonged the survival of rats bearing orthotopic glioblastomas [12]. However, the tumour-bearing rats eventually died of tumour expansion during the course of treatment and these results suggest the lumbar punctured resveratrol does not completely suppress rapid tumour growth [13].

Combinations of surgical removal and chemo- and/or radiotherapy are the standard therapeutic regimen for glioblastoma patients [14]. Although the current treatment options have somewhat improved the patient outcome, the overall prognosis of glioblastomas remains very poor due to the lack of safe and reliable adjuvant approaches to reduce toxic effects and prevent recurrence [15–17]. The evidence indicating suppressive effects of lumbar punctured resveratrol on rat orthotopic glioblastomas [12] led us to consider that the prognosis of tumour-bearing rats may be further improved if this treatment is used in combination with neurosurgery. The current study aims to address this issue using the rat orthotopic glioblastoma model employed in our previous investigations.

## Methods

### RG2 cell culture and transplantation

Rat glioblastoma RG2 cell line was kindly offered by Dr. Vencossa, Department of Neurosurgery, Central University Hospital of Lausanne (CHUV) and cultured in Dulbecco’s modified eagle medium (DMEM; Invitrogen, Grand Island, NY, USA) supplemented with 10% fetal bovine serum (Gibco Life Science, Grand Island, NY, USA) under 37 °C and 5% CO_2_ conditions.

### Ethic statement and rat orthotopic glioblastoma model

Prior to the animal experiments, the research protocols had been reviewed and approved by Animal Care and Use Committee of Dalian Medical University/DMU to guarantee that all studies involving experimental animals were performed in full compliance with National Institutes of Health Guidelines for the Care and Use of Laboratory Animals. This study used 36 male SD rats (2 months, 200 ± 20 g body weight) provided by DMU Experimental Animal Centre and raised under specific pathogen free conditions. The orthotopic glioblastoma model was established by inoculating 1 X 10^6^ RG2 cells/10 μl into the brain caudatoputamen and was followed by 2 days of analgesic and antibiotic administration [12].

### Partial tumor resection

Combination of neurosurgery with chemotherapy is commonly used to treat glioblastomas [14]. The tumour bearing rats were subjected to partial tumour resection when they suffered from intracranial hypertension and dysfunction of motion to further improve the therapeutic outcome of lumbar punctured resveratrol. The rats were anaesthetized with 10% chloral hydrate. A 2.5 mm hole was drilled in the skull with a cranial drill (RWD, 78001, Shenzhen, China) at the cell inoculation site. The electronic homemade drill was inserted into the brain to the depth of 5 mm to destroy the tumour tissue at 25,000 rpm. The damaged tumour tissue was extracted with a vacuum pump. The wound area was washed with physiological saline and the skull hole was sealed with a gelatine sponge [18]. All of the performance was undertaken under the aseptic condition. The animal general conditions were daily recorded after RG2 intracranial transplantation.

### Sample collection and treatments

The rats were sacrificed in the cold room (4 °C) painlessly by an authorized expert of DMU Animal Center via cervical dislocation by the end of the experiments or when rats suffered from paralysis, rapid weight losing and loss of appetite. The whole brains of rats were collected within 3 min. A partial tumour tissue was rapid frozen by evaporated liquid nitrogen (<-180 °C) for protein preparation and frozen section. The remanent tissue was fixed by 4% formalin, paraffin-embedded sections for immunohistochemical(IHC) and morphological examinations by the methods described elsewhere [19].

### Computer-aided tumor area measurement

The fresh brain specimens were fixed in 10% natural formalin and embedded in paraffin. The tissue-containing paraffin blocks were sectioned and then stained by hematoxylin & eosin (HE) for light microscope observation and tumor area calculation. The tumour area was measured using digital HE images of the intracranial tumours, which were saved in JPG form and opened in the Adobe Photoshop CS4 site (Adobe Systems Incorporated, San Jose, CA, USA). A 1 mm^2^ standard area unit was established in a original film/HE image, then deemed its pixel value as the numerator. The areas of all glioblastomas were simultaneously calculated through dividing every pixel value of the tumors (Y) in excel form with the pixel value of standard block (X), then multiplied by the real area of standard block (Z). The defined areas were calculated using the following formula: tumour resection rate = remaining tumour area/whole tumour area X 100%. Each of the statistical calculations were executed by more than three independent experts and the obtained data were analyzed in the method of independent-samples t-test by SSPS 17.0 (SPSS Inc., Chicago, USA). *p* < 0.05 was considered as statistical significance.

### Transmission electron microscopic examination

The freshly collected tumor specimens were rinsed with 0.1 M phosphate buffer saline (PBS; pH 7.4), immersed into 2.0% paraformaldehyde/0.1% glutaraldehyde for 90 min at 4 °C, fixed in 1% OsO_4_ for 1 h at 4 °C, dehydrated in ethanol and finally embedded in Epoxy-resin. We evaluated the ultrastructural morphometry of tissue sections by direct examination with transmission electron microscopy (JEM-2100F; JEOL, Tokyo, Japan) at 8,000× magnification. Each grid contained non-serial sections and at least 10 cells were counted [20].

### Evaluation of resveratrol-caused cellular and molecular events

Series sections in 7 μm thickness were prepared from the paraffin-embedded tumor tissues and subjected to HE staining for morphological evaluation. Immunohistochemical staining was performed on the tissue sections using a rabbit anti-Ki67 antibody (1:80; Abcam, Cambridge, UK) to evaluate proliferation activity [21]. Cells apoptosis in the tumor tissues was tested by terminal deoxynucleotide transferase(TdT)–mediated dUTP-biotin nick-end labeling (TUNEL) according to manufacturer’s instructions (Roche Diagnostics GmbH, Mannheim, Germany). STAT3 signaling as the critical survival factor of glioblastoma cells and the main molecular target of resveratrol [22, 23], the status of STAT3 signaling in the tumor tissues with and without LP resveratrol treatment was detected by Western blotting and IHC staining using rabbit anti-STAT3 antibody (1:1000 for Western blotting and 1:300 for IHC staining; Santa Cruz, CA, USA, Santa Cruz Biotech. Inc.), mouse anti-phosphorylated STAT3 antibody (1:600 for Western blotting and 1:300 for IHC staining; Chicago, IL, USA, ProteinTech Inc.) and rabbit anti-PIAS3 antibody (1:1000 for Western blotting and 1:300 for IHC staining; Santa Cruz, CA, USA, Santa Cruz Biotech. Inc.). 3,3-diaminobenzidine tetrahydrochloride (DAB, Vector Laboratories, Burlingame, CA, USA) was used as the substrate to detect binding of the primary antibody by a peroxidase reaction. Tumor tissue sections with no primary antibody incubation served as background controls for IHC staining. β-actin protein served as a quantitative control in western blot analyses.

### Statistical analysis

The data obtained from the tumour bearing rats in both groups were evaluated by the independent-samples t-test and Kaplan-Meier methods using Statistical Product and Service Solutions 17.0 software (SPSS Inc., Chicago, IL). Statistical significance was defined as *p* < 0.05. The rats was considered to be cured if their postoperative life spans were over 60 days.

## Results

### Successful partial tumor resection

**Fig. 1:**
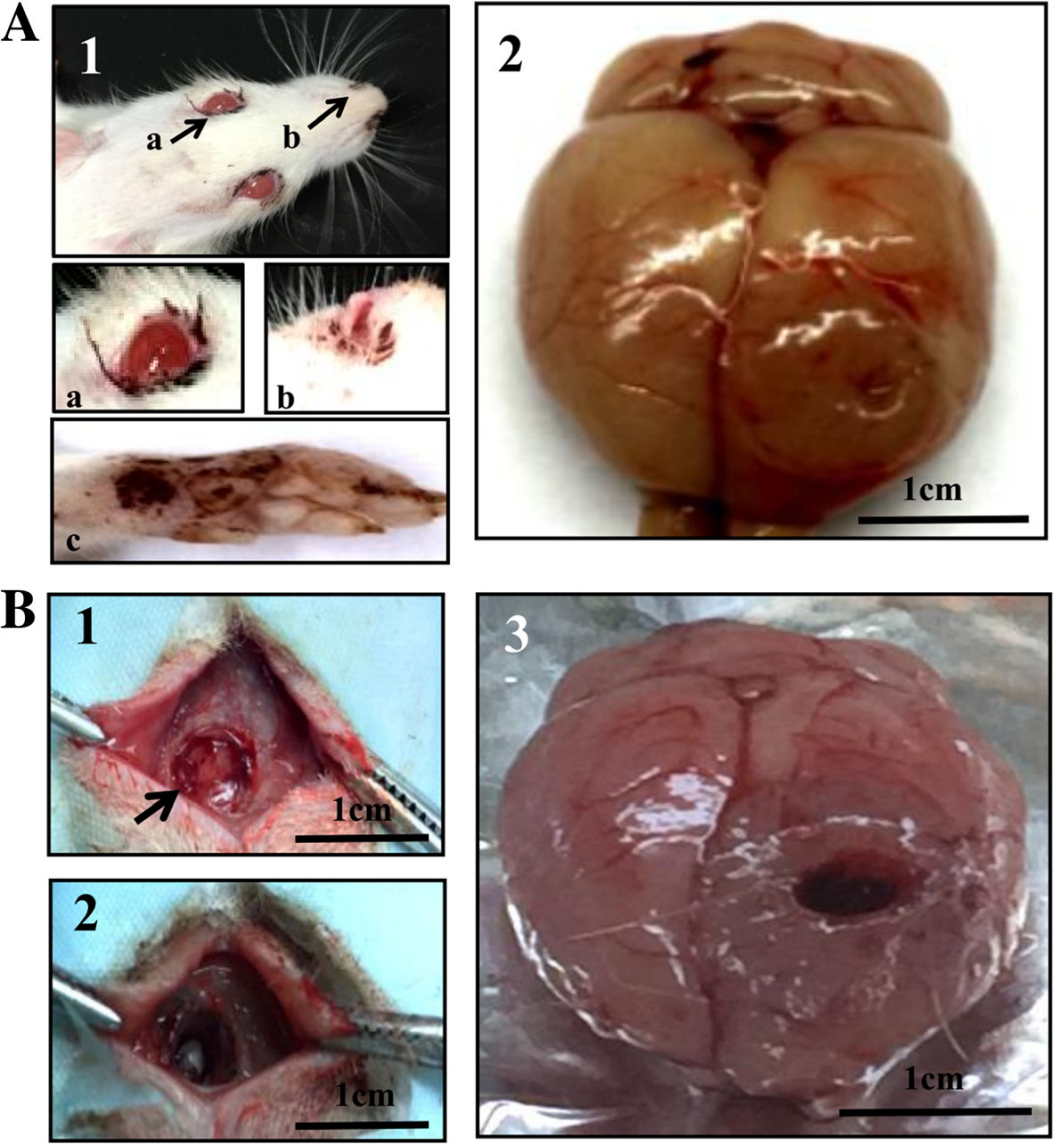
Partial resection of rat orthotopic brain tumor. **a** The onset of intracranial hypertension as surgical indication. (1) The tumour-bearing rat with binocular protrusion and peripheral eye (**a**), nasal mucosal (**b**) and the bloody claw of the same rat (**c**) after 12–18 days of orthotopic transplantation. Arrows indicate the positions shown in higher magnification. (2) The gross image of an orthotopic tumour in rat brain. **b** Brief procedures of partial tumour resection. (1) The skull was opened at the cell transplantation site to expose the tumour. The arrow indicates the craniotomy area; (2) The tumour is ablated with electric rotator at a depth of 5 mm, and the tissue was removed by vacuum suction. (3) Top view of rat brain after operation

**Fig. 2:**
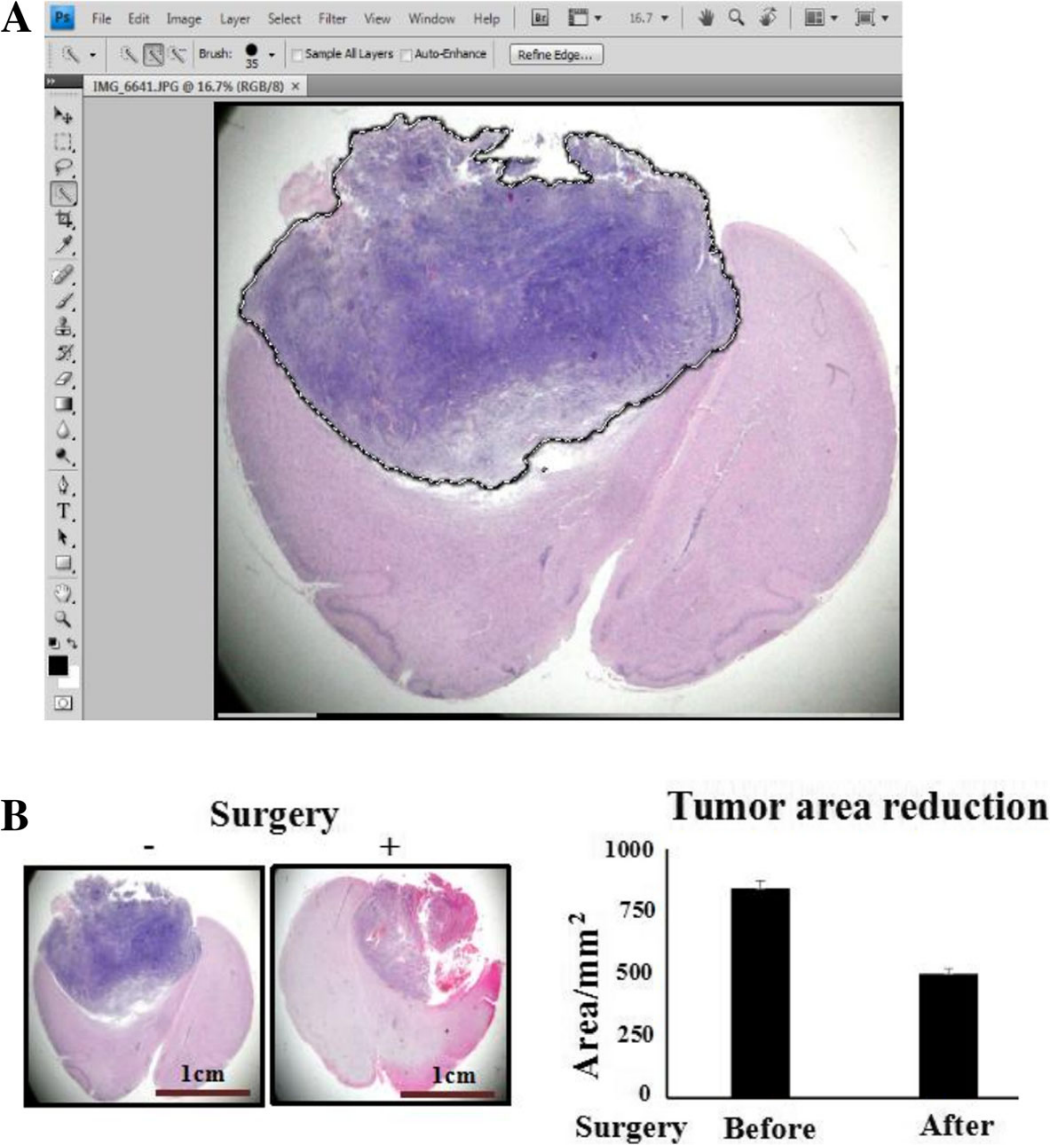
H&E image-based tumour area measurement. **a** “Magic Wand”-based approach (Adobe Photoshop CS4 site) for measuring the tumour area of the 8 rats died that died after surgery. **b** Left side: Tumour sizes before and after resection (H&E staining, X 10). Right side: Average original and postoperative tumour sizes, with statistical significance (*p* < 0.05)

In our rat model intracranial hypertension occurs at Day 12 to Day 18 after transplantation and is characterized by eye and nasal mucosal bleeding, binocular protrusion and mobility impairments (Fig. 1a). A partial tumour resection was performed on the rats with intracranial hypertension (Fig. 1b**)**. In this study, 8 of 36 tumour-bearing rats (22.2%) failed to recover from the operation because of massive haemorrhage during surgery. The remaining 28 rats (77.8%) regained full consciousness 2 h after surgery and were randomly divided into 2 experimental groups (14 rats/group): a control group without treatments and a treatment group that received LP consisting of 100 μl of 300 μM resveratrol in 3-day intervals. The brains of the deceased rats were collected to determine the tumour resection rates [24] by calculating the ratio of the tumour areas before and after resection (Fig. 2a**)**. The data showed that the average original and postoperative tumour sizes of the 8 deceased rats were 841.6 ± 31.5 mm^2^ and 499.7 ± 25.7 mm^2^, respectively (Fig. 2b). Therefore, the average resection rate (41%) of rat intracranial tumours is much lower than the resection rate (78%) observed in clinical practice [25].

### Resveratrol delivery via lumbar puncture

**Fig. 3:**
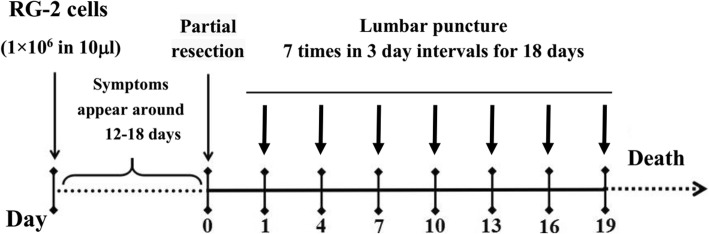
Flow diagram of surgical resection and drug administration schedule. The model rats with distinct intracranial hypertension were subjected to partial tumour resection. Lumbar puncture of 100 μl of 300 μM (6.84 μg) resveratrol started 1 day after surgery (asterisks) and was repeated in 3-day intervals for 18 days

0.228 g resveratrol (Sigma Chemical Co., St. Louis, MO, USA) was dissolved in 10 ml dimethyl sulfoxide (DMSO; Sigma Chemical Co., St. Louis, MO, USA) to prepare 100 mM stock solution. A 300 μM resveratrol working solution was prepared by mixing 3 μl of the stock solution with 1 ml physiological saline immediately prior to injection. The rats in the treatment group were anaesthetized 24 h after surgery by ether inhalation and resveratrol was lumbar punctured at the L5–6 interspace (Fig. 3) using a previously described method [12]. The total CSF volume present in the rat CNS is approximately 500 μL, so the final resveratrol concentration in CSF is approximately 50 μM [26].

### Resveratrol significantly prolonged postoperative survival times

**Fig. 4:**
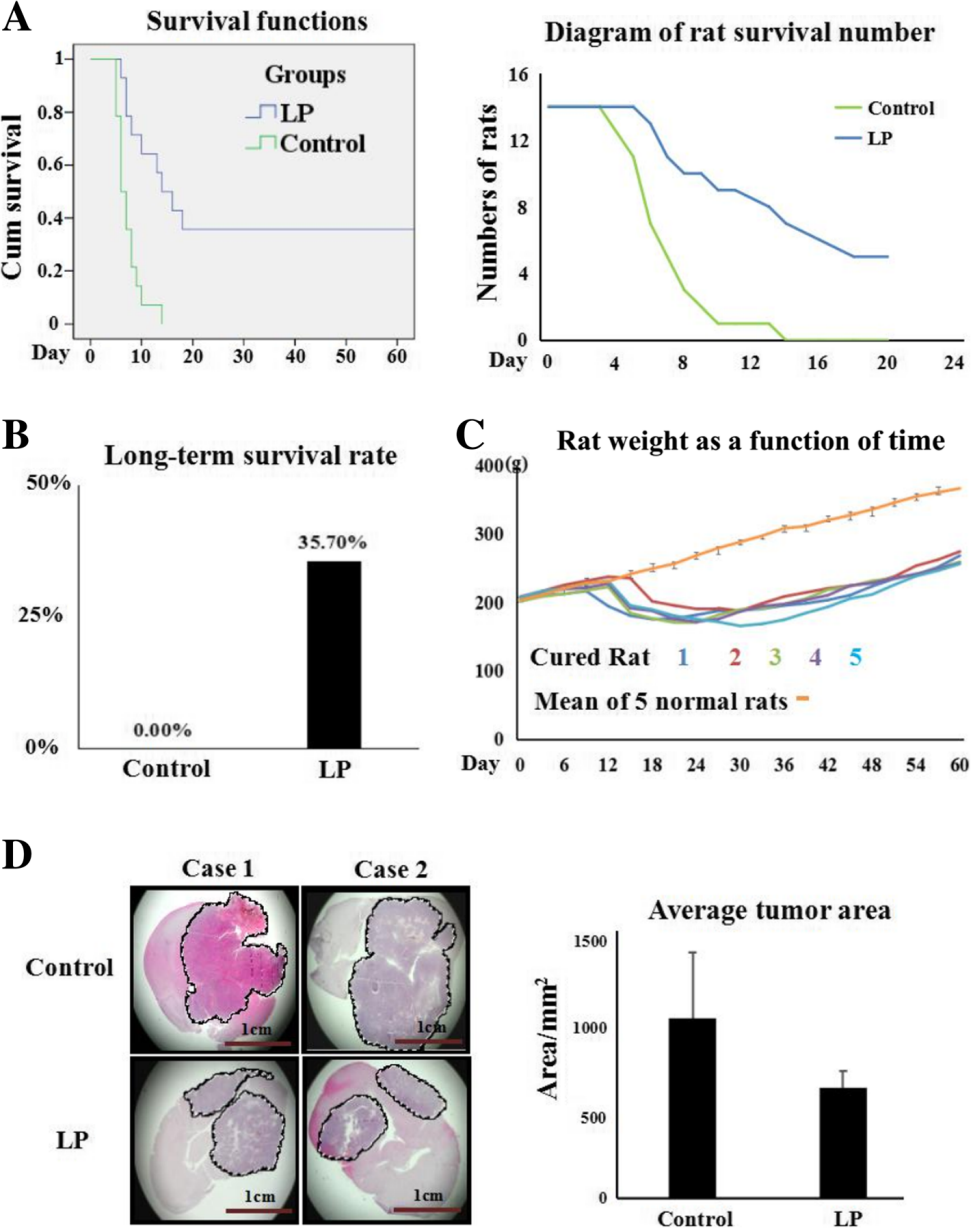
Lumbar puncture administered resveratrol inhibited tumour growth and prolonged postoperative survival time. **a** Survival curves (Left) and diagram of rat survival number (Right) of the operated rats without (control) and with LP resveratrol treatment (LP). Animal number is 14 in both groups. **b** Rate of survival rats in LP group. **c** Rat weight as a function of time. **d** H&E demonstration of tumour sizes of two cases (case 1 and case 2) of the control and LP resveratrol-treated rats died during postoperative treatment. The tumour areas were calculated 3 times for statistical analysis. Circle indicates the tumour area with dense cancer cells and strong haematoxylin staining

The postoperative survival times of the two experimental groups were recorded and compared to further ascertain the efficacy of lumbar punctured resveratrol in treating rat orthotopic glioblastomas. As shown in Fig. 4a, the control tumour-bearing animals had a mean postoperative survival time of 7.3 ± 1.3 days. Five of 14 rats in the LP group were alive over 60 days without any sign of recurrence. The mean postoperation survival time of the remaining 9 rats was 11.0 ± 2.9 days. As shown in Fig. 4b, there were significant differences in animal survival times (*p* < 0.05) and the survival rates between the control (0/14; 0%) and LP group (5/14; 35.7%). The 5 cured rats in the same batch lost weight (174.8 ± 6.4 g) before the operation, which was similar with other tumour-bearing rats. However, during the course of postoperative LP resveratrol treatment these animals gradually gained weight (273.5 ± 8.4 g). The body weights of these animals remained lower than (366.4 ± 9.3 g) the weights of normal rats in the same age range (*p* < 0.05; Fig. 4c).

### Suppressed tumour growth of LP resveratrol-treated rats

When the tumour-bearing rats were moribund, they were painlessly sacrificed and their whole brains were biopsied. The tumour area measurement was performed according to the images of the transplanted tumours without and with postoperative LP resveratrol treatment. The results revealed the average tumour area (555.8 ± 84.6 mm^2^) of LP-treated rats was smaller than (906.4 ± 330.1 mm^2^) that the control group (*p* < 0.05; Fig. 4d). These findings indicate LP resveratrol suppresses postoperative tumour growth.

### Resveratrol caused growth arrest and apoptosis

**Fig. 5:**
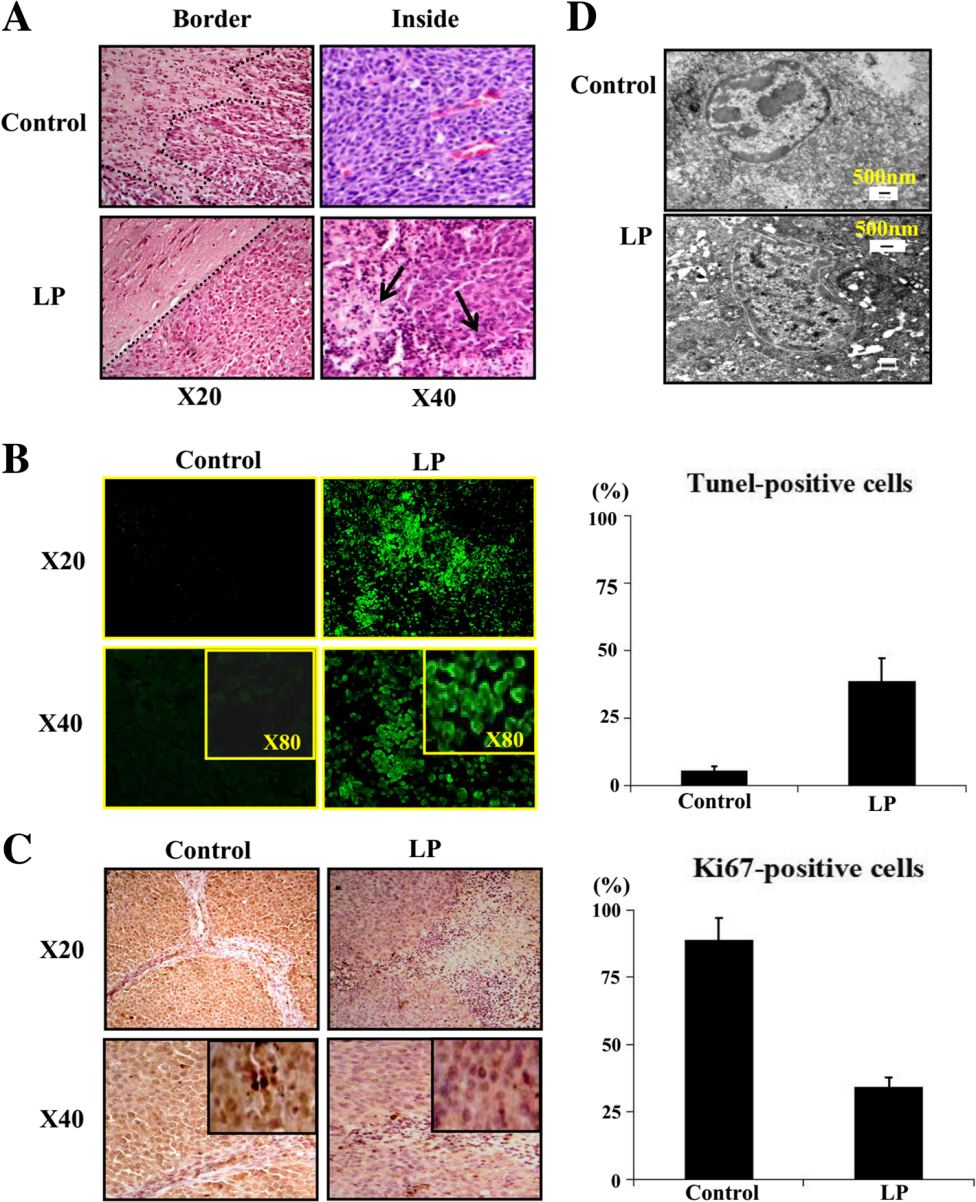
Lumbar puncture-administered resveratrol inhibits proliferation and induces apoptosis in glioblastoma cells. **a** H&E demonstration of extensive cell death caused by postoperative lumbar punctured (LP) resveratrol. The dashed line defines the tumour margin and the arrow indicates the tumour tissue with apoptosis. **b** TUNEL labelling performed on the operated tumours without (control) and with lumbar punctured resveratrol (LP). Right side, the incidences of TUNEL-positive cells in the two experimental groups. **c** Ki67 immunohistochemical staining performed on the tumours without (control) and with postoperative lumbar punctured resveratrol (LP). Right side, the incidences of Ki67-positive cells in the two experimental groups. **d** Transmission electron microscopic image of the operated tumor tissue without (Upper) and with LP resveratrol treatment (Below)

**Fig. 6:**
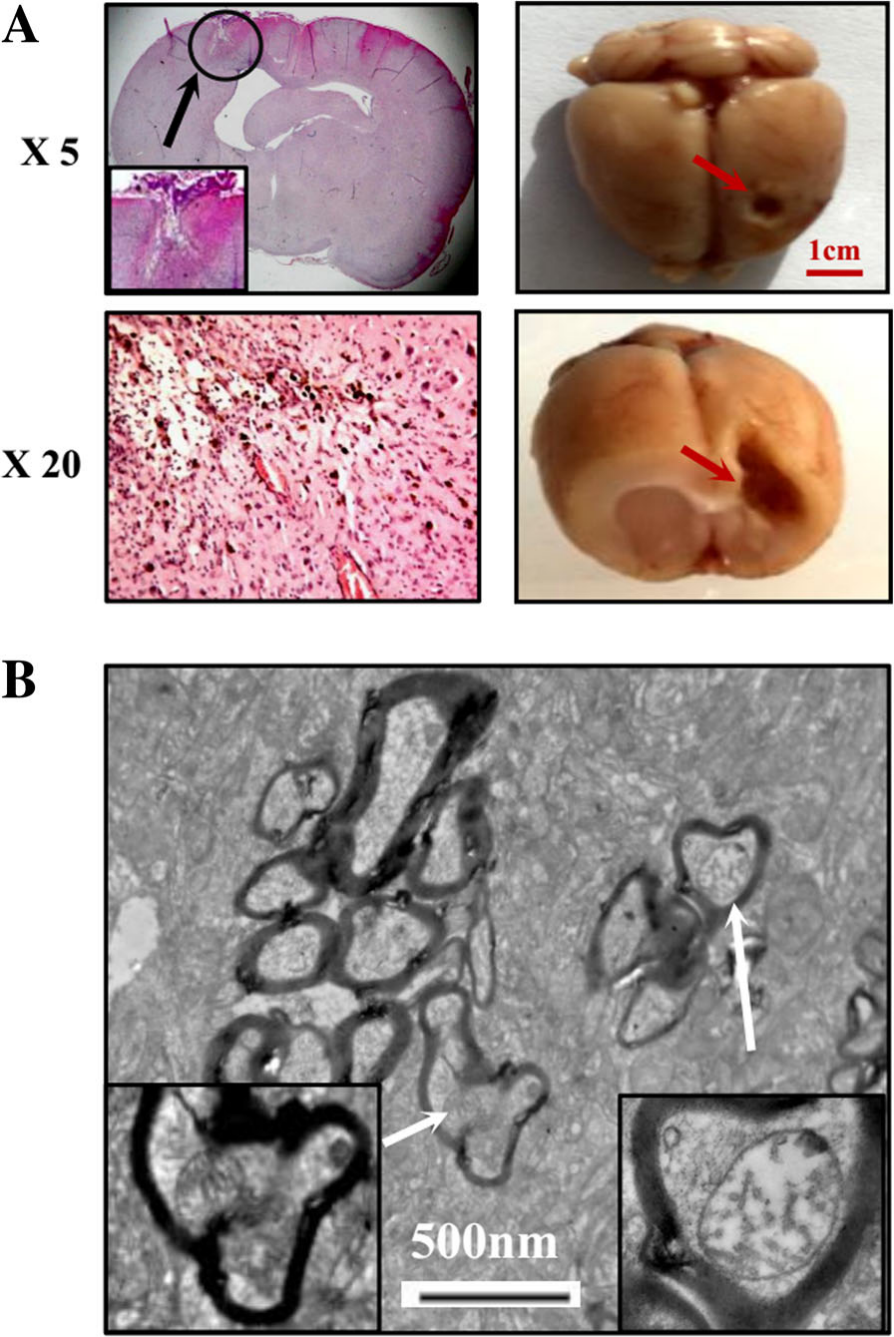
Pathological examination of the operated brain tissue of the cured rat. **a** The gross and microscopic features of the brain specimen. Arrows indicate the operated region. **b** Electron microscopic finding in the operated tissue (X 15000). Arrow indicates myelin sheaths in the insets (X 50000)

Resveratrol inhibits the in vitro growth of RG2 glioblastoma cells and causes apoptosis. Therefore, we evaluated these effects on RG2 formed orthotopic tumours. The H/E staining results demonstrated extensive cell death in the tumour tissues of LP resveratrol-treated rats, but no cell death was observed in the control group (Fig. 5a). A small cavity surrounded by gliofibrosis was observed in the operated region of the five cured rats (Fig. 6a). The TUNEL assay results demonstrated the apoptotic regions were widely distributed in the tumour tissues of the LP group but staining was uncommon in their control counterparts (Fig. 5b). Additionally, the apoptosis rate was significantly reduced in the control group compared to the LP group (*p* < 0.05). The immunohistochemical staining showed decreased frequencies of Ki67-positive cells in resveratrol-treated tumours (Fig. 5c; *p* < 0.05). Cumulatively, our data indicate resveratrol caused growth arrest and apoptosis in the operated tumours.

### Ultra-structural evidenced apoptosis

To further confirm resveratrol-caused apoptotic death, transmission electron microscopic examination was conducted on the corresponding tumor tissues examined by TUNEL assay. The results revealed that no ultra-structural alteration was observed in the tumour samples of the control group. In contrast, cell shrinkage and apoptotic body formation were commonly observed in the LP resveratrol treated tumour tissues (Fig. 5d). The results showed that in place of the glioblastoma cells the tissues were rich in mitochondria-containing myelin sheaths in the operated brain regions of the LP resveratrol cured rats (Fig. 6b), which indicates their cancer-free status and neurological reconstruction [27].

### Resveratrol regulates STAT3 signalling and PIAS3 expression

**Fig. 7:**
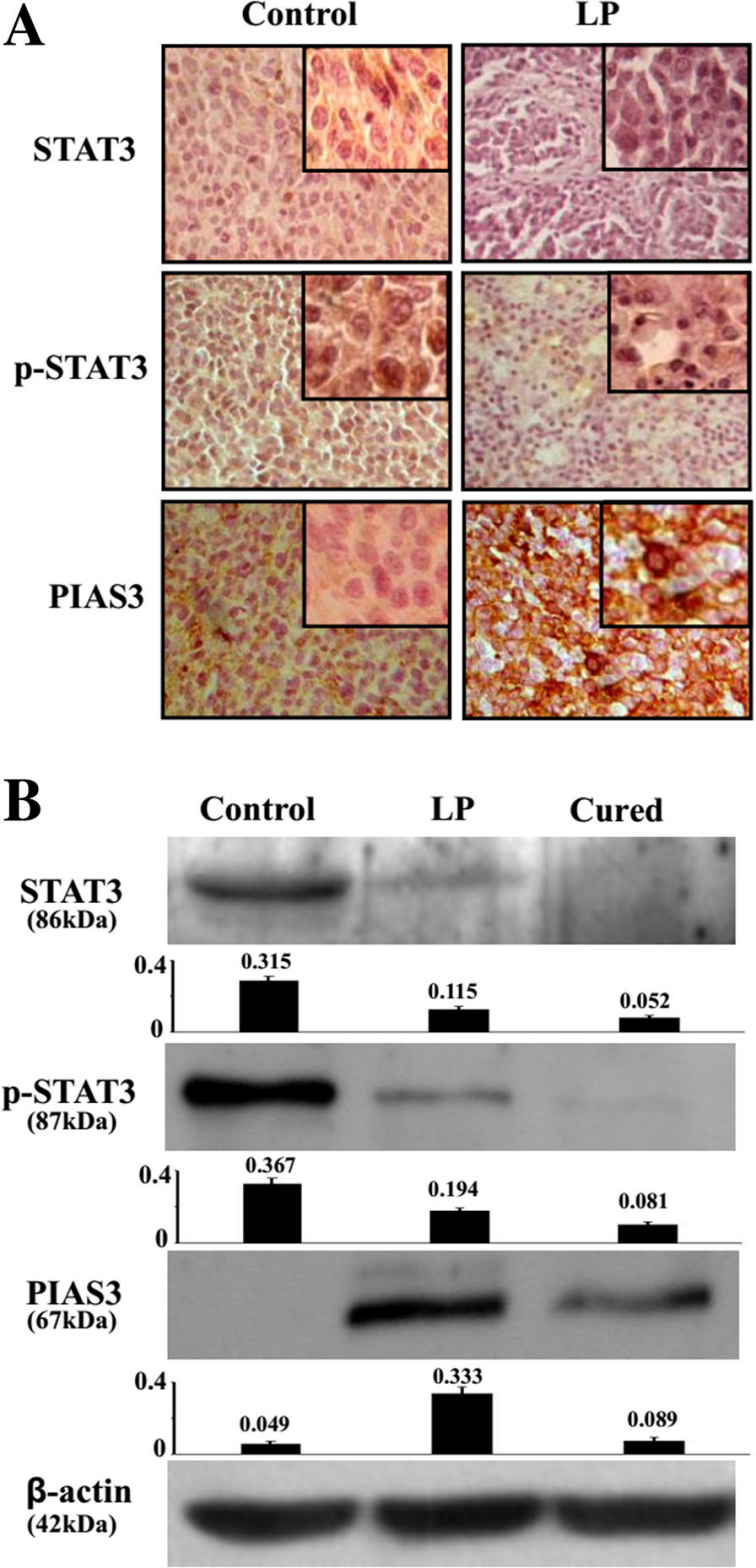
Evaluation of STAT3 signaling and PIAS3 expression in the operated tumor tissues without (Control) and with LP resveratrol treatment (LP) as well as the brain tissue of the cured rat by immunohistochemical staining (**a**) and Western blotting (**b**)

p-STAT3-oriented immunohistochemistry was performed on tissue microarrays constructed with the brain tumors and surrounding tissues of the animals with and without resveratrol treatment. The results showed STAT3 and p-STAT3 were undetectable in normal brain tissues. However, both were highly expressed in untreated glioblastoma tissues and were remarkably decreased in resveratrol-treated tumours, particularly in the regions with extensive cell death. The level of the STAT3 signalling inhibitor PIAS3 was increased in resveratrol-treated tumours of the LP group (Fig. 7a). Western blotting (Fig. 7b) further demonstrated the reduction of STAT3 (63.5%) and p-STAT3 levels (47.1%) and up-regulation of PIAS3 expression (580.3%) in the tumors treated by LP resveratrol (*p* < 0.05). These results demonstrated the capacity of resveratrol to inactivate STAT3 signaling and to promote PIAS3 expression in vivo.

## Discussion

The main challenges in glioblastoma treatment are tumour recurrence due to the difficulty of radical tumour resection and the severe adverse effects of conventional anticancer drugs [28]. Although trans-resveratrol possesses anti-glioblastoma activity without neurotoxic effects [12], the amount of systemic resveratrol in the brain is extremely low due to the efficient enzymatic bio-transforming system in normal cells [13]. Lumbar puncture/LP is a chemotherapeutic approach to treat brain malignancies such as central nervous system leukemia (CNS-L), because of its easy performance and organ-targeted drug delivery [29]. Our data from rat experimental models have demonstrated that LP administration remarkably increases intracranial resveratrol concentrations and prolongs the average tumour-bearing time from 16.0 ± 1.8 days to 22.2 ± 2.1 days [12]. Although these results are promising, the rats treated eventually died of tumour expansion and this suggests resveratrol alone is unable to halt orthotopic glioblastoma growth. The therapeutic outcome of LP resveratrol needs to be further improved through combinations with other conventional anti-glioblastoma strategies [30].

In the clinical management of glioblastomas, surgery is the first choice to remove extensive tumour tissue (approximately 78%), followed by postoperative chemo- and/or radio-therapy to prevent tumour relapse [25]. We mimicked this therapeutic regimen by using partial resection (41% average) of the orthotopic glioblastomas when the animals displayed intracranial hypertension followed by resveratrol lumbar puncture administration in regular intervals [12]. The mean postoperative survival time (28.5 ± 12.9 days) of 14 LP resveratrol-treated rats was significantly longer than that of the control (7.3 ± 1.3 days) animals treated with surgery alone. Moreover, five rats in the LP group were alive for over 60 days and showed recovered general conditions without any sign of recurrence. The detailed reason(s) leading to the different fates (death and survival) of the rats in LP group is currently unclear. The prolonged life span (44.5 ± 14.7 days) and the five cured cases in this group suggest better therapeutic outcome of the combined approach than treatment by neurosurgery (23.3 ± 3.1 days) or LP resveratrol only (22.2 ± 2.1 days) [12]. The effectiveness of this combined therapy might be applicable to glioblastoma patients because the resection of their tumours is more radical than our rat partial resection model.

The increasing intracranial hypertension due to continued tumour expansion is the main cause of the animal death, which can be reflected by exophthalmos, peripheral bleeding, dyskinesia and hemiplegia [31, 32]. In our model, the animals will die within 2 days of symptom appearance. Therefore, partial tumour resection was conducted to release the pressure and then the operated rats were randomly separated into the groups without and with LP resveratrol treatment in two-day intervals. The animals were sacrificed when they reached the agonal stage and the tumour sizes/areas were calculated. When we exclude the 5 cured rats, the average tumour area of the remaining 9 rats in LP group is smaller (555.8 ± 84.6 mm^2^) than the area (906.4 ± 330.1 mm^2^; *p* < 0.05) of the untreated group. These findings further confirm the efficiency of postoperative LP resveratrol in suppressing the outgrowth of the tumour. However, the animals in the LP group died before the tumours grew as large as those in the control group. This phenomenon indicated the general anaesthesia used at each LP resveratrol administration may affect rat health and shorten their life span. In clinical practice, local anaesthesia is used for lumbar puncture [33]. Therefore, the adverse effects of repeated general anaesthesia on rat health do not apply to the patients.

A body of evidence reveals that resveratrol exerts multiple anticancer effects on glioblastoma cells, including growth inhibition and apoptosis induction [34]. STAT3 signaling is critical for glioblastoma cells including RG2 cells and is the major molecular target of resveratrol [35, 36]. To elucidate cellular and molecular events caused by postoperative LP resveratrol treatment, the ultra-structural features, the level of Ki67 expression, the status of STAT3 signaling and its negative regulator/PIAS3 expression in the tumor tissues of the control and resveratrol-treated group were examined [37, 38]. The results revealed the untreated tumour tissues showed aggressive growth with high levels of Ki67 expression and limited apoptotic cells. Conversely, clearer tumour border, decreased Ki67 labelling frequency and widely distributed TUNEL+ cells with ultra-structural features of apoptosis were observed in LP resveratrol treated tumour tissues. The reduction of STAT3 expression and p-STAT3 nuclear translocation accompanied with PIAS3 upregulation was observed in LP resveratrol treated rats and not in the control specimens. Importantly, we did not observe morphological alterations in the noncancerous brain tissues, and there were no disabling dyskinesias in the 5 cured rats. These findings provide strong cellular and molecular evidence of the effectiveness and safety of this combined approach in the in vivo treatment of advanced orthotopic glioblastomas.

## Conclusion

This study demonstrates that the combination of lumbar puncture resveratrol administration with partial tumour resection significantly prolongs the mean survival time of glioblastoma rats and 35.7% (5/14) of the treated rats are cured. The tumour tissues in the combination treatment group showed growth inhibition, extensive apoptosis, suppressed STAT3 expression and nuclear translocation and upregulated PIAS3 expression. Our findings demonstrate postoperative resveratrol administration through lumbar puncture efficiently improves the prognosis of rat advanced orthotopic glioblastoma and could be a potential option for the management of glioblastomas.
